# Fractional Flow Reserve in the Diagnosis of Ischemic Heart Disease in a Patient with Coronary Artery Ectasia

**DOI:** 10.3390/diagnostics12010017

**Published:** 2021-12-23

**Authors:** Malgorzata Zalewska-Adamiec, Lukasz Kuzma, Hanna Bachorzewska-Gajewska, Slawomir Dobrzycki

**Affiliations:** 1 Department of Invasive Cardiology, Medical University of Bialystok, 15276 Bialystok, Poland; kuzma.lukasz@gmail.com (L.K.); hana.bachorzewska-gajewska@umb.edu.pl (H.B.-G.); slawomir.dobrzycki@umb.edu.pl (S.D.); 2 Department of Clinical Medicine, Medical University of Bialystok, 24A Sklodowskiej-Curie St., 15276 Bialystok, Poland

**Keywords:** coronary artery ectasia, coronary artery disease, coronary artery aneurysm, fractional flow reserve, coronarography

## Abstract

Coronary artery ectasias (CAE) are diffuse dilatations of coronary artery segments with a diameter 1.5 times greater than the largest adjacent normal segment of the vessel. They are found in 0.3–5.0% of coronary angiography. Risk factors for CAE include atherosclerosis, previous percutaneous coronary interventions, arterial inflammation and connective tissue diseases. The diagnosis of CEA in a patient is a considerable diagnostic and therapeutic problem due to the unfavorable prognosis and the lack of guidelines. We present a case of a 69-year-old male patient with a history of retrosternal pain admitted to the clinic for the diagnosis of coronary artery disease. In coronary angiography, numerous ectases of the main coronary arteries and atherosclerotic lesions causing border stenosis of the left anterior descending (LAD), diagonal (2D) and marginal branch (OM). The heart team decided to assess the significance of the changes with the fractional flow reserve (FFR). The FFR was performed and haemodynamically insignificant stenoses of the ectatically dilated coronary arteries were found. The patient was qualified for conservative treatment.

**Figure 1:**
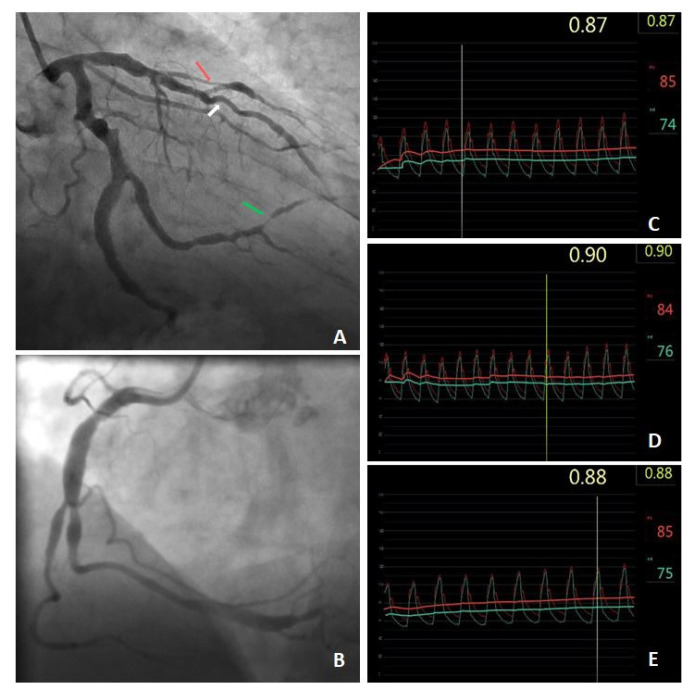
A 69-year-old male patient with arterial hypertension and hypercholesterolaemia, with a one-year history of stress angina and positive electrocardiographic exercise test, was admitted to the clinic for invasive diagnosis. The patient underwent coronary angiography which showed numerous ectasias of the coronary arteries with atherosclerotic lesions; LAD ectatically widened to half its length with 50% stenosis in the middle segment (white arrow), 70% stenosis in the mouth of 2D (red arrow), Cx (circumflex artery) ectatically altered along its entire length, 1OM ectatically widened with 75% stenosis in the distal segment (green arrow) and RCA (right coronary artery) changed ectatically to half length (**A**,**B**). The heart team qualified the patient for the assessment of the significance of stenoses in LAD and 2D by FFR (fractional flow reserve). FFR measurement was performed according to the current practical guide; hemodynamically insignificant stenosis was found in LAD (**C**), 2D (**D**) and OM (**E**). The patient was qualified for further conservative treatment of the coronary artery disease and trimetazidine was added to pharmacotherapy. After 4 months, the patient feels well and does not report retrosternal pain. In the presented case, apart from atherosclerotic lesions, the patient was diagnosed with numerous ectases of the main coronary arteries, i.e., the most advanced type 1 according to the Markis classification (this figure) [1,2,3]. Slow flow within the ectatically dilated segments of the coronary arteries and the presence of significant atherosclerotic stenosis increase the risk of myocardial ischemia [1,4]. In this case, the FFR value is limited due to possible microcirculation disturbances and measurement errors caused by abnormal blood flow within the CEA. To extend coronary physiological evaluation, coronary flow reserve (CFR) measurement, and flow separation index (FSi) calculation can give detailed information about the microvascular coronary status, and can provide an additive value in the functional assessment of ectasia and other slow coronary flow situations [5,6]. Patients with CEA were most often excluded from clinical trials and so far no guidelines cover ectasy of the coronary arteries, so making decisions about the optimal treatment of these patients is extremely difficult.

## Data Availability

Original data supporting the reported results are available through contacting the authors.
